# Silver nanoclusters with Ag^2+/3+^ oxidative states are a new highly effective tool against phytopathogenic bacteria

**DOI:** 10.1007/s00253-023-12596-z

**Published:** 2023-06-08

**Authors:** Benedetta Orfei, Chiaraluce Moretti, Stefania Loreti, Giuseppe Tatulli, Andrea Onofri, Luca Scotti, Antonio Aceto, Roberto Buonaurio

**Affiliations:** 1 Department of Agricultural, Food and Environmental Sciences, University of Perugia, Perugia, Italy; 2 Council for Agricultural Research and Economics (CREA), Research Centre for Plant Protection and Certification, Roma, Italy; 3 Department of Medical, Oral and Biotechnological Sciences, “G. d’Annunzio” University of Chieti-Pescara, Chieti, Italy

**Keywords:** Silver nanoparticles, *Pseudomonas syringae* pv. *tomato*, *Xanthomonas vesicatoria*, *Xylella fastidiosa* subsp. *pauca*, *Clavibacter michiganensis* subsp. *michiganensis*

## Abstract

**Abstract:**

The main measure worldwide adopted to manage plant bacterial diseases is based on the application of copper compounds, which are often partially efficacious for the frequent appearance of copper-resistant bacterial strains and have raised concerns for their toxicity to the environment and humans. Therefore, there is an increasing need to develop new environmentally friendly, efficient, and reliable strategies for controlling plant bacterial diseases, and among them, the use of nanoparticles seems promising. The present study aimed to evaluate the feasibility of protecting plants against attacks of gram-negative and gram-positive phytopathogenic bacteria by using electrochemically synthesized silver ultra nanoclusters (ARGIRIUM‑SUNCs^®^) with an average size of 1.79 nm and characterized by rare oxidative states (Ag^2+/3+^). ARGIRIUM‑SUNCs strongly inhibited the in vitro growth (effective concentration, EC_50_, less than 1 ppm) and biofilm formation of *Pseudomonas syringae* pv. *tomato* and of quarantine bacteria *Xanthomonas vesicatoria*, *Xylella fastidiosa* subsp. *pauca*, and *Clavibacter michiganensis* subsp. *michiganensis*. In addition, treatments with ARGIRIUM‑SUNCs also provoked the eradication of biofilm for *P. syringae* pv. *tomato*, *X. vesicatoria*, and *C. michiganensis* subsp. *michiganensis.* Treatment of tomato plants via root absorption with ARGIRIUM‑SUNCs (10 ppm) is not phytotoxic and protected (80%) the plants against *P. syringae* pv. *tomato* attacks. ARGIRIUM‑SUNCs at low doses induced hormetic effects on *P. syringae* pv. *tomato*, *X. vesicatoria*, and *C. michiganensis* subsp. *michiganensis* as well as on tomato root growth. The use of ARGIRIUM‑SUNCs in protecting plants against phytopathogenic bacteria is a possible alternative control measure.

**Key points:**

*• ARGIRIUM‑SUNC has strong antimicrobial activities against phytopathogenic bacteria;*

*• ARGIRIUM‑SUNC inhibits biofilm formation at low doses;*

*• ARGIRIUM‑SUNC protects tomato plants against bacterial speck disease.*

## Introduction

Plant diseases caused by phytopathogenic bacteria place major constraints on crop production and cause significant annual losses on a global scale, estimated at over $1 billion dollars (Mansfield et al. 2012; Kannan et al. 2015). This scenario gets worse and worse in response to increased global commerce and climate change, which will make plant diseases, including those caused by bacteria, more frequent and severe (Ristaino et al. 2021; IPPC Secretariat 2021). The recent introduction in Europe of the quarantine bacterium *Xylella fastidiosa* is an example of how the expansion of international trade in agriculture has facilitated the spread of a dangerous pathogen. In southern Italy, *X. fastidiosa* subsp. *pauca*, the agent of the olive quick decline syndrome, has destroyed millions of olive trees and has profoundly modified the landscape typically characterized by centenary olive trees (Saponari et al. 2019; IPPC Secretariat 2021). There is evidence that the introduction of *X. fastidiosa* in Europe has taken place through infected ornamental coffee plants originated from Central America and shipped to Europe (Scortichini 2022). Furthermore, it is likely that climate changes will affect the future distribution of *X. fastidiosa* as predicted by the models built by Bosso et al. (2016) and Godefroid et al. (2022).

All plant bacterial diseases are difficult to manage, and a combination of control measures is required to contrast a given bacterial disease. The main measure adopted worldwide for controlling these diseases is the application of copper compounds, which are often partially efficacious as they do not penetrate into the plants, where the phytopathogenic bacteria provoke infections, and for the frequent appearance of copper-resistant bacterial strains (Sundin et al. 2016; Fan et al. 2022). In addition, the high level of Cu accumulation in the soil, the risk of surface and sub-surface water contamination, and potential public health problems due to Cu entering the food chain have raised concerns on the use of Cu in agriculture (Lamichhane et al. 2018). Consequently, there is worldwide regulatory pressure on agriculture in general, especially in organic production, to restrict the use of these compounds. For example, the European Commission has included Cu compounds in the list of substances as candidates for substitution. Therefore, there is an increasing need to develop new environmentally friendly, efficient, and reliable strategies for controlling bacterial diseases, and among them, the use of nanotechnology (Wang et al. 2016) and in particular nanoparticles (NPs) seems promising (Sundin et al. 2016; Elmer and White 2018; Balestra and Fortunati 2022). NPs are currently defined as any material that has one or more dimensions at the scale of 1 to 100 nm (Sekhon 2014), and those tested in plant protection are termed nanopesticides or nano plant protection products (Kookana et al. 2014). Nanopesticides could offer a range of benefits including increased efficacy, durability, and a reduction in the amounts of active ingredients that need to be used. There is increasing evidence that metal-based NPs have antimicrobial in vitro activity against phytopathogenic bacteria and are able to protect plants from bacterial diseases (Sundin et al. 2016; Li et al. 2022).

Pompilio et al. (2018) have electrochemically synthesized silver ultra nanoclusters, registered as ARGIRIUM‑SUNCs^®^, with polygonal shape and a particularly small average size (1.79 nm) and which have antibacterial and anti-biofilm activities against human pathogenic bacteria at concentrations ≤ 1 ppm (Molina-Hernandez et al. 2021). ARGIRIUM‑SUNCs characterization by X-ray diffraction and XPS analysis revealed the presence of Ag_3_O_4_ and AgO crystalline phase corresponding to Ag^+^, Ag^2+^, and Ag^3+^ silver oxides on the surface of the and Ag° in the core (Gasbarri et al. 2021). The presence in the stable form of these rare oxidation states of silver represents a main point of novelty of this formulation. As a consequence, ARGIRIUM‑SUNCs are characterized by a negative solvation shell (zeta potential values in the range − 40/ − 70 mV). This high absolute zeta potential value suggests that nanoparticles, surrounded by anionic solvation, tend to repulse each other, avoiding any aggregation process. This explains the long stability (> 1 year) of the solution, as well as the stabilization effect of double bonds present in geometric stereoisomers (Angelini et al. 2019). Due to the presence of Ag^2+^ and Ag^3+^, the oxidation–reduction potential (ORP) resulted very high, + 400 mV in comparison to − 90 mV obtained from the dithiothreitol (DTT) reducing agent standard solution (Thermo Scientific™, Waltham, MA, USA).

The objectives of the present study were to investigate in vitro the antimicrobial and anti-biofilm activities of ARGIRIUM‑SUNCs against four phytopathogenic bacteria, three gram-negative: *Pseudomonas syringae* pv. *tomato*, *Xanthomonas vesicatoria*, and *X. fastidiosa* subsp. *pauca*, and one gram-positive: *Clavibacter michiganensis* subsp. *michiganensis*. The last three bacteria are included in the list A2 of the quarantine pests (EPPO Global Database; www.eppo.int/ACTIVITIES/plant_quarantine/A2_list). These bacteria provoke the following economically important plant diseases: tomato bacterial speck (*P. syringae* pv. *tomato*), tomato bacterial spot (*X. vesicatoria*), tomato bacterial canker (*C. michiganensis* subsp. *michiganensis*), and olive quick decline syndrome (*X. fastidiosa*). Another objective was to evaluate in vivo the efficacy of ARGIRIUM‑SUNCs in protecting tomato plants against *P. syringae* pv. *tomato*.

## Materials and methods

### Nanoparticles generation

Silver ultra nanoclusters (ARGIRIUM‑SUNCs^®^) were electrochemically synthesized by using an improved synthetic protocol in ultra-pure water without stabilizing agents or other chemical components as previously reported (Scotti et al. 2017; Pompilio et al. 2018; Grande et al. 2020; Molina‑Hernandez et al. 2021; Borgolte et al. 2022). The synthesis method is protected by the European patent: EP-18181873.3. The physicochemical properties of ARGIRIUM-SUNCs, as determined by transmission electron microscope (TEM), X-ray electron scanning microscopy (XR-SEM), X-ray diffraction (XRD), X-ray photoelectron spectroscopy (XPS), matrix-assisted laser desorption/ionization time-of-flight (MALDI TOF), dynamic light scattering (DLS), ultraviolet–visible spectrophotometry (UV–Vis), ion selective electrode (ISE), are: a very small size, 1.79 nm ± 1.004 (ultra nano cluster), (Z-potential), plasmon resonance spectrum (λ max at 410 nm) and nonspherical shape, stability at acidic pH (2–12) (Gasbarri et al. 2019).

### Bacterial strains and culture conditions

The gram-negative bacterial species *P. syringae* pv. *tomato* DAPP-PG 215 (LMG 33003), *X. vesicatoria* DAPP-PG 466 (LMG 33004) and *X. fastidiosa* subsp. *pauca* strain De Donno (CFBP 8402) and the gram-positive bacterium *C. michiganensis* subsp. *michiganensis* CM2 (LMG 3690) were used in the present study. All bacterial strains were stored at − 80 °C in 15% glycerol. *X. fastidiosa* subsp. *pauca* was grown on PD2 broth medium (Davis et al. 1980) at 28 °C, while the other bacterial species were on nutrient agar (NA; Thermo Fisher Scientific, Waltham, MA, USA) at 27 °C.

### In vitro ARGIRIUM‑SUNCs antimicrobial activity

The effect of ARGIRIUM‑SUNCs on *P. syringae* pv. *tomato*, *X. vesicatoria*, and *C. michiganensis* subsp. *michiganensis* growth was evaluated in vitro by measuring changes in optical density using the Thermo Scientific MultiSkan EX microplate reader (Thermo Fisher Scientific, Waltham, MA, USA) and 96-well ELISA microplates. Briefly, bacteria were grown on NA plates for 24 h at 27 °C and then resuspended in King’s Broth (KB; King et al. 1954) medium. Bacterial suspensions were spectrophotometrically adjusted at a concentration of 10^8^ CFU (colony-forming unit) mL^−1^ (OD_660_ nm = 0.06). Each well of the ELISA microplate was filled with 80 µL of KB medium, 100 µL of ARGIRIUM-SUNCs at different concentrations, and 20 µL of bacterial suspension. The ranges of concentrations established in preliminary experiments were: 0.1–1.0 ppm for *P. syringae* pv. *tomato* and *X. vesicatoria*, and 0–6 ppm for *C. michiganensis* subsp. *michiganensis.* The microplate reader, placed in a growth chamber at 25 °C, was set to shake the plate every minute for 5 s and to make a reading every h for 24 h at OD_630nm_ or every 10 min for 6 h for exploring the first phase of the growth.

*X. fastidiosa* subsp. *pauca* was grown in PD2 agar medium for 20 days at 28 °C, scraped off, resuspended in PD2 broth and grown to 10^8^ CFU mL^−1^. The bacterial concentration was spectrophotometrically measured at A_600_ = 0.8 OD; bacterial inoculum (60 μL) was transferred in 6 mL of PD2 broth alone, as control, or in PD2 broth supplemented with ARGIRIUM‑SUNCs.

The efficacy of ARGIRIUM‑SUNCs on *X. fastidiosa* subsp. *pauca* growth was evaluated both measuring changes in optical density and bacterial DNA by qPCR according to Francis et al. (2006) using SYBR select master mix for CFX96 Real-Time System (Applied Biosystem, Foster City, CA, USA).

Genomic DNA was extracted from each bacterial culture (500 μL) using the Gentra Puregene Yeast/Bact. Kit (Qiagen, PL Venlo, The Netherlands) according to the manufacturer’s instructions for gram-negative bacteria.

### Effect of ARGIRIUM‑SUNCs on bacterial biofilm formation and disruption

The effect of ARGIRIUM‑SUNCs on biofilm formation was investigated using the crystal violet assay as essentially indicated by Pompilio et al. (2018). *P. syringae* pv. *tomato*, *X. vesicatoria*, and *C. michiganensis* subsp. *michiganensis* were grown in KB at 27 °C for 24–48 h in the presence of increasing doses of ARGIRIUM‑SUNCs, ranging from 0.1 to 1.0 ppm, while *X. fastidiosa* subsp. *pauca* De Donno was grown in PD2 agar medium at 28 °C for 15 days in the presence of ARGIRIUM‑SUNCs 0.023, 0.23, or 2.3 ppm.

The effect of ARGIRIUM‑SUNCs on *P. syringae* pv. *tomato*, *X. vesicatoria*, and *C. michiganensis* subsp. *michiganensis* biofilm formation was investigated using the crystal violet assay as essentially indicated by Pompilio et al. (2018), while *X. fastidiosa* subsp. *pauca* biofilm formation was evaluated following Zaini et al. (2009) and modified as reported by Baldassarre et al. (2020).

The effect of ARGIRIUM‑SUNCs on biofilm disruption was evaluated in *P. syringae* pv. *tomato*, *X. vesicatoria*, and *C. michiganensis* subsp. *michiganensis* as described below. Ten-mL borosilicate glass tubes filled with 1.8 mL of KB medium and 200 µL of the bacterial suspensions (10^8^ CFU mL^−1^) were incubated for 48 h at 27 °C in the dark. After 48 h, to the tubes washed three times with sterile water to remove non-adherent bacteria were added 2 mL of KB medium containing ARGIRIUM-SUNCs to the final concentrations of 1 × minimum inhibitory concentration (MIC), 2 × MIC, and 4 × MIC. After a further 24 h of incubation, tubes were rinsed three times with sterile water, filled with 2.5 mL of 0.1% crystal violet for 20 min for staining under static conditions at room temperature, washed three more times with sterile water, and then let dry. Finally, 2.5 mL of ethanol was added to the tubes to allow the elution of crystal violet. After 30 min, the absorbance was read at 630 nm.

### Plant material and ARGIRIUM‑SUNCs phytotoxicity measurement

Seeds of tomato plants (*Solanum lycopersicum* L., cv. Rio Grande) were grown in a seedbed containing modular tray substrate (Klasmann-Deilmann, GmbH; Geeste, Germany), and 10–14 days after sowing, the seedlings were transplanted in (9 × 9 × 12.5 cm) plastic pot (1 plant per pot) containing the above-mentioned substrate. Before and after the transplant, plants were kept in a growth chamber programmed for a 12-h day at 25 °C and 12-h night at 18 °C with 70–90% RH. White fluorescent lamps provided 240 µE m^−2^ s^−1^ illumination.

Phytotoxicity of ARGIRIUM‑SUNCs was evaluated in tomato leaves and roots. The leaves of tomato plants at the 3^rd^ true leaf stage were sprayed until the run-off with ARGIRIUM-SUNCs at concentrations ranging from 0 to 10 ppm, and the appearance of toxicity symptoms was daily evaluated up to 10 days post-treatment.

The phytotoxicity on roots was evaluated by determining the level of cell death using the method described by Schützendübel et al. (2001) with slight modifications. Tomato seeds were set to germinate in wet filter paper for 5 days in the dark at 27 °C. Four excised tomato roots (50–60 mg of fresh weight) per sample were transferred in 15 mL-plastic tubes containing 0–15 ppm of ARGIRIUM‑SUNCs and incubated for 24 h in the dark. After incubation, the roots were gently washed three times (5 min for each washing) in deionized water and immersed for 1 h in a 0.25% Evans blue solution. To induce a high level of cell death, some root samples immersed and incubated in water were kept in a water bath at 80 °C for 20 min and stained with Evans blue as above reported. After several washing with water to remove the dye, roots were homogenized in a mortar with 1 mL solution of methanol (50% v/v) and 1% sodium dodecyl sulfate (SDS; w/v) and successively incubated in a water bath at 50 °C for 15 min. After centrifugation at 14,000 g for 15 min, the absorbance at 600 nm of the supernatants was read.

### Effect of ARGIRIUM‑SUNCs on tomato root growth

To optimize the modality of application ARGIRIUM‑SUNCs to tomato plants, we explored the possibility of seed applications. Since we preliminary observed that ARGIRIUM‑SUNCs stimulated the root growth during seed germination, this prompted us to plan experiments to study this phenomenon. Tomato seeds were set to germinate in filter paper immersed in ARGIRIUM‑SUNCs at concentrations ranging from 0 to 10 ppm for 8 days in the dark at 27 °C, and the root length was measured.

### Effect of ARGIRIUM‑SUNCs in protecting tomato plants against *P. syringae pv. tomato*

To verify whether ARGIRIUM‑SUNCs was able to protect tomato plants against *P. syringae* pv. *tomato*, the nanomaterial was applied by spraying the leaves or dipping the roots. For leaf application, leaves of tomato plants at the 3^rd^–4^th^ true leaf stage were sprayed until the run-off with ARGIRIUM‑SUNCs at 10 ppm or with deionized sterile water as control. For root application, tomato plants at the 3^rd^ true leaf stage were taken out of their substrate, their roots gently and carefully washed with tap water to remove the substrate, and placed in a glass container filled with 200 mL of ARGIRIUM‑SUNCs 10 ppm or 200 mL of deionized sterile water (control). Plants immersed in the ARGIRIUM‑SUNCs or water were kept in the growth chamber for 4 days or 8 days. At the end of the exposure period, each plant was transplanted into pots containing the above-mentioned substrate.

Two days after the foliar treatments or 6–10 days after root treatments with ARGIRIUM-SUNCs, the leaves of the treated and untreated plants were spray-inoculated with a suspension (10^8^ CFU mL^−1^) of *P. syringae* pv. *tomato* and kept for the first 48 h under a plastic bag to maintain a high level of relative humidity. Plants were maintained in a growth chamber programmed at 22 ± 2 °C under a 12-h light/12-h dark cycle with 70 ± 90% RH and with 240 µE m^−2^ s^−1^ light illumination. Fourteen days after the inoculation, images of treated and untreated leaves were captured with a Nikon D90 digital camera (Nikon Europe B.V., Amstelveen, The Netherlands), and disease severity, expressed as a percentage (%) of infected leaf area, was evaluated on the three distal leaflets of the 4^th^ and 5^th^ leaves using Assess: Image Analysis Software for Plant Disease Quantification (APS Press, St. Paul, MN, USA; Lamari 2002).

### Statistical analyses

Experiments were repeated three times with three replicates per treatment and values expressed as means ± standard error. Data obtained from the antimicrobial activity of ARGIRIUM‑SUNCs on the growth of *P. syringae* pv. *tomato*, *X. vesicatoria*, and *C. michiganensis* subsp. *michiganensis* were submitted to non-linear regression analysis based on the log-logistic dose-response model proposed by Streibig and Rudemo (1993), which allowed the calculation of the effective concentration (EC) and to plot the dose-response curves for each bacterial species.

Data about tomato root length as affected by the ARGIRIUM‑SUNCs doses in a non-phytotoxic range were also analyzed by using non-linear regression (Fig. 6B), based on the hormetic equation proposed by Brain and Cousens (1989):

1 $$y=c+ \frac{d-c+fx}{1+exp\left\{b\left[\mathrm{log}\left(x\right)-\mathrm{log}(e)\right]\right\}}$$

where *y* is the root length, *x* is the dose, *c* is the response at a very high dose, *b* is the slope near to the inflection point, *d* is the response at the zero dose (control), *e* is the abscissa at the inflection point, and *f* is the parameter measuring the hormetic effect. When *f* is equal to zero, the above equation reduces to the previously mentioned log-logistic (non-hormetic) dose–response model.

Data about the effect of low doses of ARGIRIUM‑SUNCs (effective concentration, EC_10_, and EC_20_) on the early growth of *P. syringae* pv. *tomato*, *X. vesicatoria*, and *C. michiganensis* subsp. *michiganensis* were submitted to non-linear regression based on the equation:

2 $$y=b{X}^{c}+a$$

where the response *y* represents the OD_630_ changes in the first 6 h of growth (every 10 min), *X* is the time, *a* is the OD_630_ at time = 0, *b* is the growth rate, and *c* describes the eventual presence of the lag phase in the growth curve. In detail, when *c* is higher than 1, the growth curve is characterized by an initial lag phase, while when *c* is equal to 1, the growth curve is linear with no initial lag phase.

For all regression models, the goodness of fit was assessed by graphical analyses of residuals and approximate *F*-tests for lack of fit.

All the other data were subject to one-way (treatment) analysis of variance (ANOVA); pairwise comparisons of means were performed by using Duncan’s multiple range tests at *p* < 0.05.

Statistical analyses were performed by using the Excel^®^ software (Microsoft Office 2013; Milano, Italy), with the aid of the macros BIOASSAY97 and DSAASTAT (Onofri and Pannacci 2014; Perugia, Italy).

## Results

### ARGIRIUM‑SUNCs have a strong antibacterial activity

**Fig. 1:**
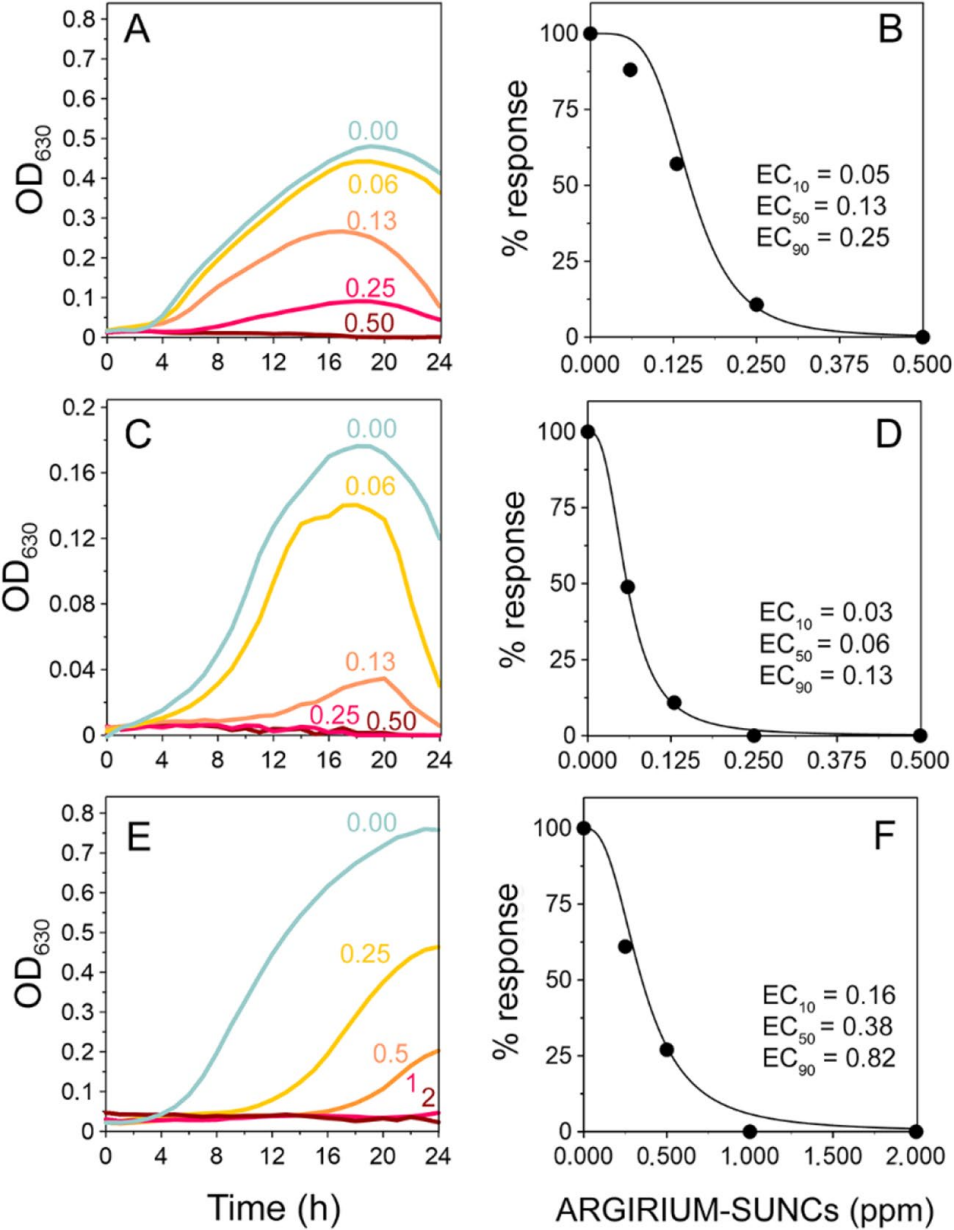
In vitro growth curves of *Pseudomonas syringae* pv. *tomato* DAPP-PG 215 (**A**), *Xanthomonas vesicatoria* DAPP-PG 466 (**C**), and *Clavibacter michiganensis* subsp. *michiganensis* CM2 (E) in the presence of different doses of ARGIRIUM-SUNCs. Dose–response curve for *P. syringae* pv. *tomato* (**B**), *X. vesicatoria* (**D**), and *C. michiganensis* subsp. *michiganensis* (**F**) calculated at 24 h after the ARGIRIUM-SUNC treatments. Values reported inside each plot represent ARGIRIUM-SUNC concentrations expressed in ppm. Effective concentrations were indicated in the plot

**Fig. 2:**
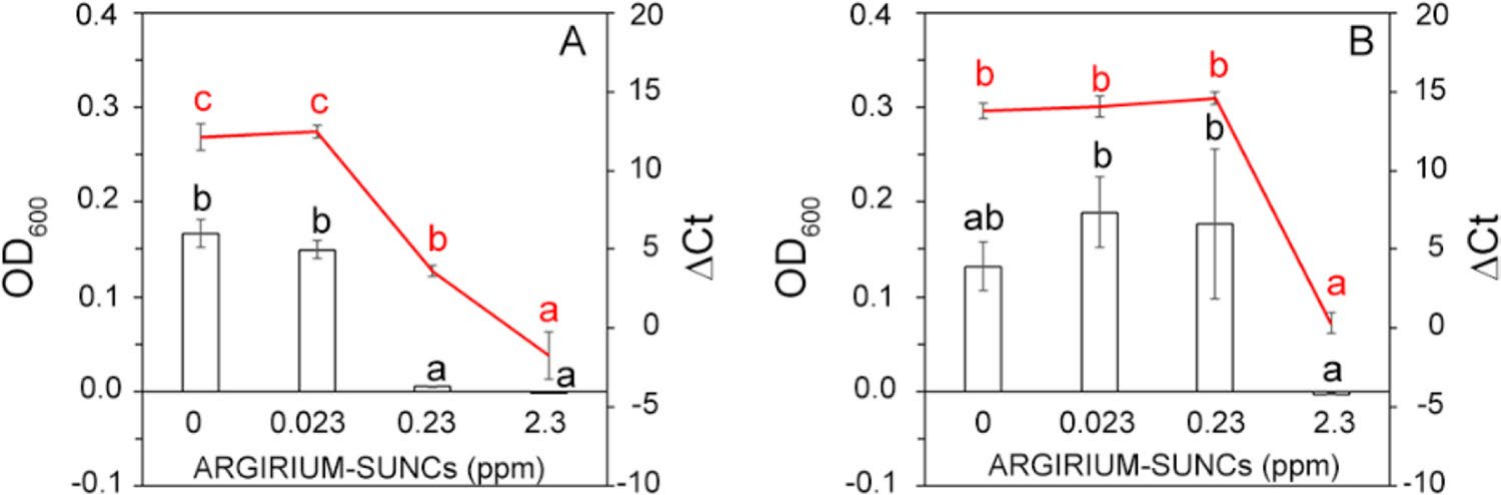
Effect of ARGIRIUM-SUNCs at different concentrations on the growth of *Xylella fastidiosa* subsp. *pauca* De Donno after 6 (**A**) and 15 (**B**) days; bacterial growth was expressed as OD_600_ (columns) or assessed by qPCR on *X. fastidiosa* subsp. *pauca* DNA (red line). ∆Ct represents the difference between the Ct value obtained at 6 (**A**) and 15 (**B**) days of growth and the Ct value at time 0 for each sample

ARGIRIUM‑SUNCs at concentrations lower than 1 ppm inhibited in vitro growth of both gram-negative (*P. syringae* pv. *tomato*, *X. vesicatoria*, and *X. fastidiosa* subsp. *pauca*) and gram-positive (*C. michiganensis* subsp. *michiganensis*) bacteria assayed (Fig. 1). Dose-response curves (Eq. 1, with *f* = 0) showed a good fit to the observed and were used to calculate EC_50_ values of 0.13, 0.06, and 0.38 ppm for *P. syringae* pv. *tomato*, *X. vesicatoria*, and *C. michiganensis* subsp. *michiganensis*, respectively (Fig. 1). Marked and statistically significant reductions in *X. fastidiosa* subsp. *pauca* growth were recorded 6 and 15 days after treatment with ARGIRIUM‑SUNCs at 2.3 ppm; the reduction was also significant at 0.23 ppm only 6 days after the treatment (Fig. 2). These results were confirmed with *X. fastidiosa* subsp. *pauca* DNA concentration changes determinations (Fig. 2).

### Low doses of ARGIRIUM‑SUNCs stimulate bacteria in the early phase of growth

**Fig. 3:**
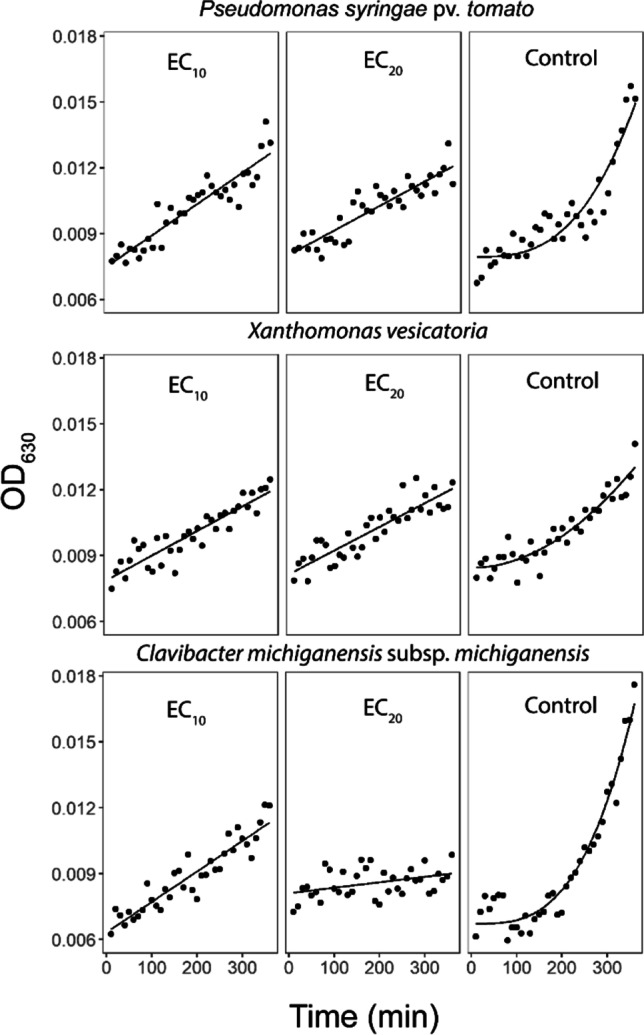
Effect of low ARGIRIUM-SUNC concentrations (EC_10_ and EC_20_) on the early growth (first 6 h) of *Pseudomonas syringae* pv. *tomato* DAPP-PG 215, *Xanthomonas vesicatoria* DAPP-PG 466, and *Clavibacter michiganensis* subsp. *michiganensis* CM2

The effect of low doses of ARGIRIUM‑SUNCs (EC_10_ and EC_20_) on the early growth of *P. syringae* pv. *tomato*, *X. vesicatoria*, and *C. michiganensis* subsp. *michiganensis* was described in Fig. 3 by using Eq. 2. As reported in Table 1, the values of *c* are statistically > 1 when *P. syringae* pv. *tomato*, *X. vesicatoria*, and *C. michiganensis* subsp. *michiganensis* are grown in the absence of ARGIRIUM‑SUNCs (controls). By contrast, when bacteria were treated with ARGIRIUM‑SUNCs at low doses, the *c* values are not statistically different from 1 indicating that the treatments stimulate *P. syringae* pv. *tomato*, *X. vesicatoria*, and *C. michiganensis* subsp. *michiganensis* to grow just after a few minutes without an initial lag phase.

**Table 1:** Effect of low doses of ARGIRIUM-SUNCs (AS) on the early bacterial growth of *Pseudomonas syringae* pv. *tomato*, *Xanthomonas vesicatoria*, and *Clavibacter michiganensis* subsp. *michiganensis*

Bacteria	Treatments	*c* values ± SE^a^	*t*-test (c = 1)	*p*-values
*P. syringae* pv. *tomato*	Control	2.953 ± 0.167	11.705**	0.0000
	AS (EC_10_)	1.062 ± 0.240	0.260	0.7964
	AS (EC_20_)	0.971 ± 0.249	− 0.117	0.9076
*X. vesicatoria*	Control	1.952 ± 0.320	2.978**	0.0054
	AS (EC_10_)	1.578 ± 0.355	1.625	0.1136
	AS (EC_20_)	1.002 ± 0.289	0.008	0.9932
*C. michiganensis* subsp. *michiganensis*	Control	2.936 ± 0.256	7.570**	0.0000
	AS (EC_10_)	1.436 ± 0.279	1.561	0.1278
	AS (EC_20_)	0.995 ± 0.263	− 0.019	0.9849

^a^*c*-value is the exponent of Eq. 2: when *c* is > 1 the curve presents an exponential trend with an initial lag phase, while when *c* = 1 the growth is linear without an initial lag phase. In the equation, *Y* is the OD_630_, *X* is the time, a describe the OD_630_ at zero time, and *b* is the growth rate. *SE*, standard error

** value of *t*-test statistically significant at *p* < 0.01

### ARGIRIUM‑SUNCs inhibit biofilm formation and determine biofilm eradication

**Fig. 4:**
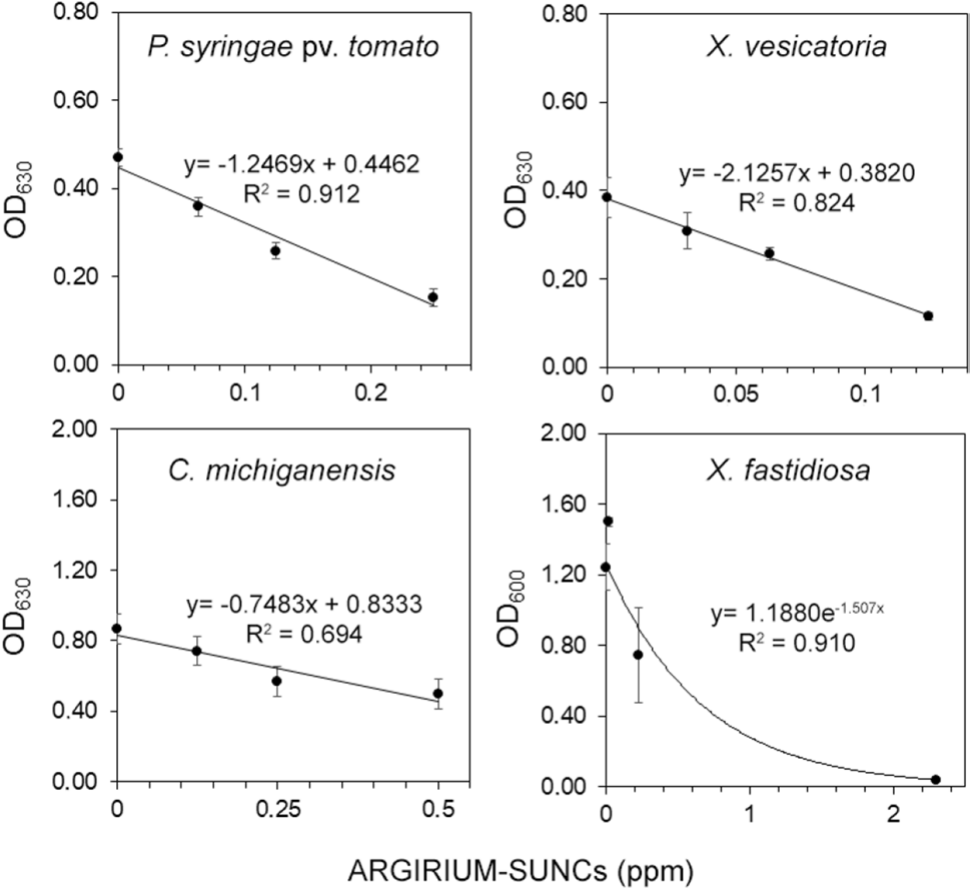
Effect of different doses of ARGIRIUM-SUNCs on biofilm formation in *Pseudomonas syringae* pv. *tomato* DAPP-PG 215, *Xanthomonas vesicatoria* DAPP-PG 466, *Clavibacter michiganensis* subsp. *michiganensis* CM2, and *Xylella fastidiosa* subsp. *pauca*, De Donno (CFBP 8402). Symbols represent the observed data together with the fitted line

As reported in Fig. 4, significant linear decreasing relationships between increasing doses of ARGIRIUM‑SUNCs and biofilm formation were observed 48 h after the treatments for *P. syringae* pv. *tomato*, *X. vesicatoria*, and *C. michiganensis* subsp. *michiganensis* and after 15 days for *X. fastidiosa* subsp. *pauca*. At the maximum concentration assayed, the reductions in the biofilm formation were 67, 70, 43, and 97% for *P. syringae* pv. *tomato*, *X. vesicatoria*, and *C. michiganensis* subsp. *michiganensis* and *X. fastidiosa* subsp. *pauca*, respectively (Fig. 4).

**Fig. 5:**
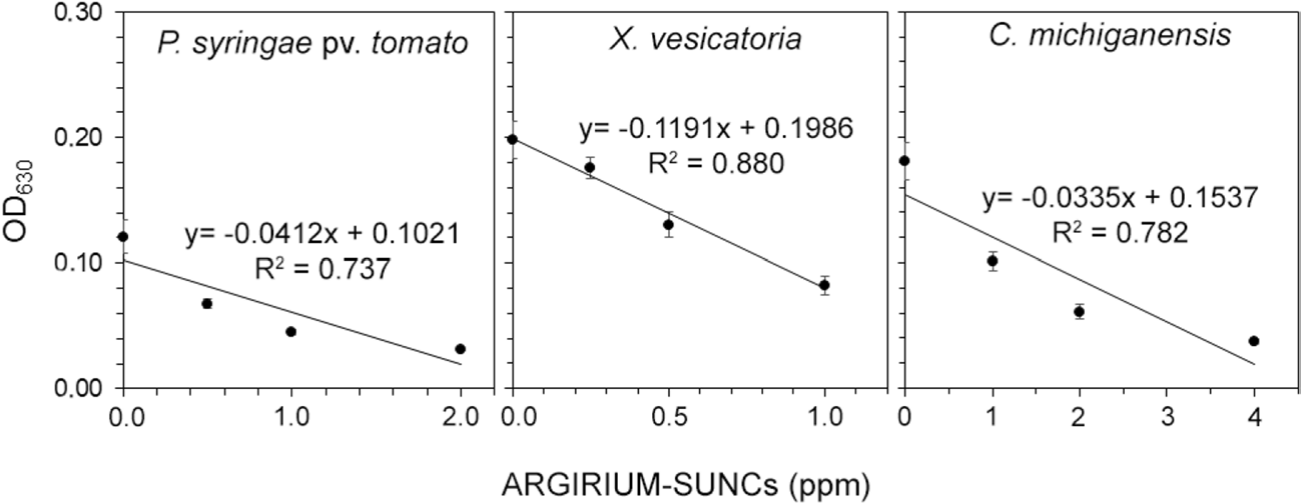
Effect of different doses of ARGIRIUM-SUNCs on biofilm disruption in *Pseudomonas syringae* pv. *tomato* DAPP-PG 215, *Xanthomonas vesicatoria* DAPP-PG 466, and *Clavibacter michiganensis* subsp. *michiganensis* CM2. Biofilm disruption was evaluated 24 h after the treatment with 1 × MIC, 2 × MIC, and 4 × MIC of ARGIRIUM-SUNCs. Symbols represent the observed data together with the fitted line

Treatments with ARGIRIUM‑SUNCs also provoked the eradication of biofilm for *P. syringae* pv. *tomato*, *X. vesicatoria*, and *C. michiganensis* subsp. *michiganensis* (Fig. 5). In fact, significant linear decreasing relationships between increasing doses of ARGIRIUM‑SUNCs and biofilm eradication were recorded (Fig. 5). At the maximum concentration assayed, the reductions in the biofilm formation were 74, 58, and 79% for *P. syringae* pv. *tomato*, *X. vesicatoria*, and *C. michiganensis* subsp. *michiganensis*, respectively (Fig. 5).

### ARGIRIUM‑SUNCs have a dual effect on tomato plants

**Fig. 6:**
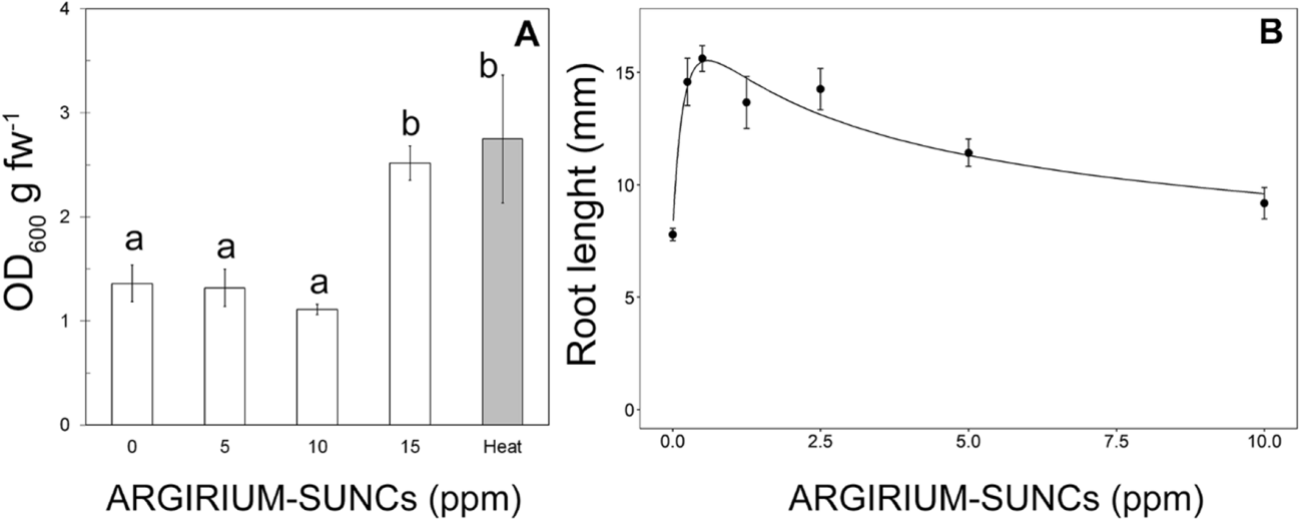
Assays carried out on tomato roots to evaluate the toxicity of ARGIRIUM-SUNCs (**A**) and the effect of this nanomaterial on root growth (**B**). **A** Effect of the treatments of tomato roots with different doses of ARGIRIUM-SUNCs on cell death as determined by Evans blue method. As further control (heat) of a high level of cell death, tomato roots were immersed in a water bath at 80 °C for 20 min. **B** Effect of non-phytotoxic doses of ARGIRIUM-SUNCs on root length (mm) of tomato seedlings 9 days after the treatments

ARGIRIUM‑SUNCs were toxic at concentrations ≥ 15 ppm on tomato roots and stimulated (hormetic effect) the growth of tomato roots during seed germination at concentrations ≤ 10 ppm (Fig. 6A).

In fact, when tomato roots were treated with increasing doses of ARGIRIUM‑SUNCs, cell death, determined with the Evans blue staining, was observed only in roots treated with 15 ppm of ARGIRIUM‑SUNCs and not at concentrations ≤ 10 ppm (Fig. 6A). The level of cell death at 15 ppm of ARGIRIUM‑SUNCs was similar to those observed in heat-treating roots (Fig. 6A).

Equation 1 showed a good fit to the observed data and clearly demonstrated that low doses of ARGIRIUM‑SUNCs stimulate the growth of tomato roots (e.g., twice the control value at 0.5 ppm) (Fig. 6B), as the estimated parameter *f*, which measures the hormetic effect, is significantly different from zero.

### ARGIRIUM‑SUNCs applied to the roots protect tomato plants against *P. syringae pv. tomato*

**Fig. 7:**
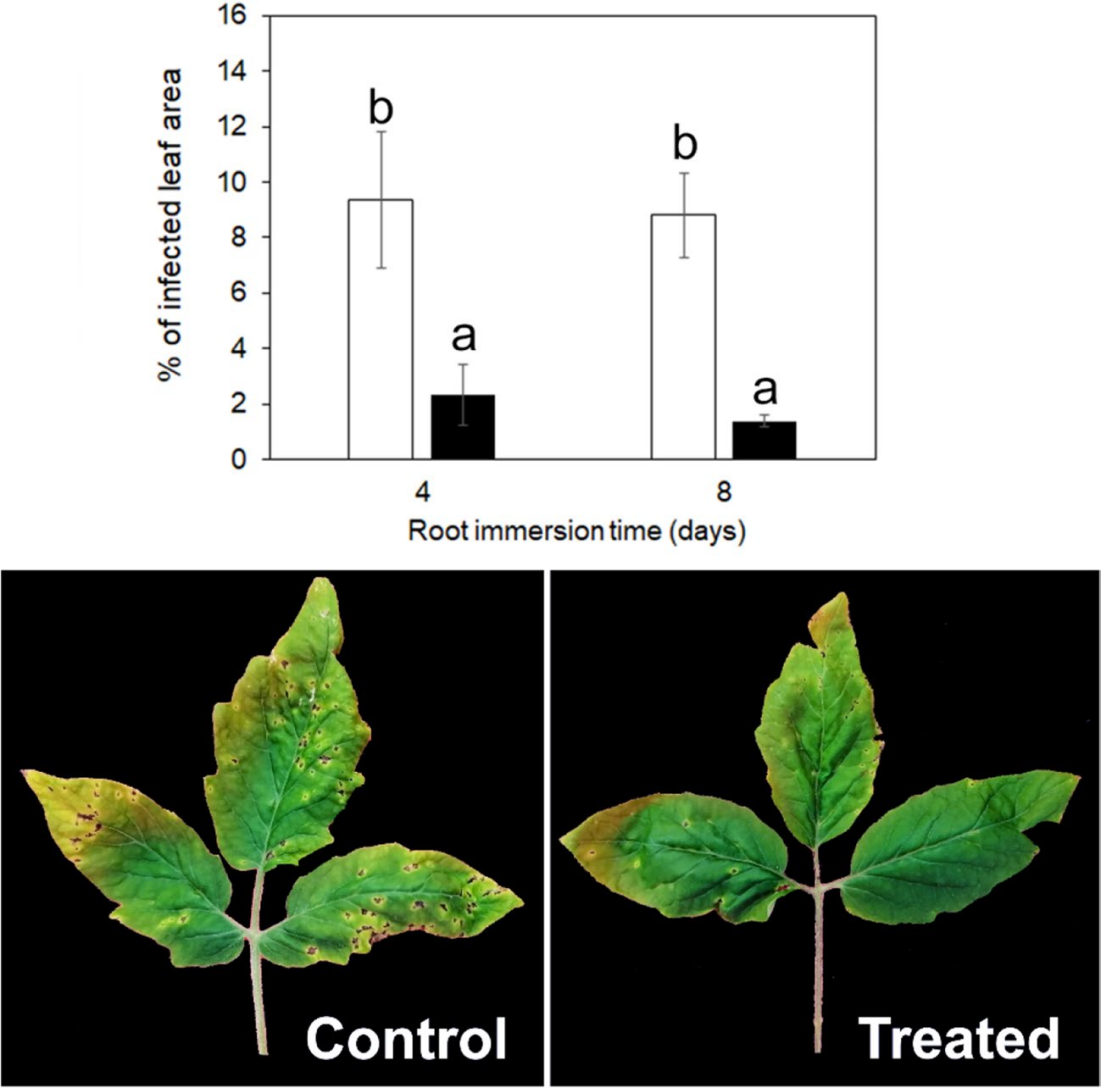
The plot reports the effects of treatment of tomato plants with 10 ppm ARGIRIUM-SUNCs, by root uptake for 4 and 8 days, on disease severity caused by *Pseudomonas syringae* pv. *tomato* DAPP-PG 215, 14 days after the inoculation. Empty columns = untreated control plants; filled columns = ARGIRIUM-SUNCs treated plants. The image shows the protective effect of ARGIRIUM-SUNCs (8 days of root uptake) on the three distal leaflets of the 4th tomato leaves against *Pseudomonas syringae* pv. *tomato*, 14 days after the inoculation

Spray application of ARGIRIUM‑SUNCs to tomato leaves did not protect tomato plants against *P. syringae* pv. *tomato*, while root application significantly protected them (Fig. 7). In fact, tomato plants treated with 10 ppm of ARGIRIUM‑SUNCs absorbed by roots for 4 or 8 days were protected at 75 and 84% against *P. syringae* pv. *tomato* attacks, respectively (Fig. 7).

## Discussion

Current management strategies to control plant bacterial diseases are based on a number of measures including improved cultural practices; the application of bactericides, plant activators, and biocontrol agents; and the use of resistant plant cultivars when available (Sharma et al. 2022). However, effective management remains a challenge as the longevity of deployed measures is threatened by constantly changing bacterial populations (Sharma et al. 2022). A durable and sustainable strategies for controlling bacterial diseases could be the use of nanoparticles (Sundin et al. 2016; Li et al. 2022).

Among various inorganic and organic nanomaterials, silver nanoparticles (AgNPs) have attracted the attentions of many researchers for their well-documented antimicrobial activity (Tariq et al. 2022), and they have been used in many pesticides registered in recent years (Bergeson 2010; Gogos et al. 2012). The antimicrobial activity of silver has long been known and exploited in medicine, and it is due to the release of silver ions. However, due to the ability to form complexes, silver ions exerted their activity for only a short time, a disadvantage overcome by the use of AgNPs (Francesconi et al. 2022).

We here demonstrated that AgNPs and in particular ARGIRIUM‑SUNCs strongly inhibited the in vitro growth of the phytopathogenic bacteria *P. syringae* pv. *tomato*, *X. vesicatoria*, *C. michiganensis* subsp. *michiganensis*, and *X. fastidiosa* subsp. *pauca* at doses less than 1 ppm. Our results are similar to those obtained by Molina-Hernandez et al. (2021) against human pathogenic bacteria. The AgNPs tested against phytopathogenic bacteria, described in the literature, are larger in size and exhibit antimicrobial activity at higher doses compared to ARGIRIUM‑SUNCs (Tariq et al. 2022). For example, the AgNPs used by Ibrahim et al. (2019) against *Xanthomonas oryzae* pv. *oryzae* and *Acidovorax oryzae* have a size of 25–50 nm and an antimicrobial activity at 20 ppm.

It is known that AgNPs with small sizes favor the interaction with the bacterial membranes and are more toxic (Morones et al. 2005; Lu et al. 2013). Besides, smaller nanoparticles exhibit a higher surface area that may also enhance dissolution (Xiu et al. 2012; Dobias and Bernier-Latmani 2013; Mitrano et al. 2014). Furthermore, the high efficacy of ARGIRIUM‑SUNCs could be due to their greater purity as it was synthesized in the absence of contaminants, such as inorganic or organic agents, commonly used as stabilizers in other preparations (Kittler et al. 2010).

The lower antimicrobial activity of ARGIRIUM‑SUNCs we found against the gram-positive bacterium *C. michiganensis* subsp. *michiganensis* respect to the gram-negative ones is in agreement with data reported in the literature on AgNPs (Tariq et al. 2022). This difference in action might be attributed to the difference in the cell wall composition of gram-positive—particularly in peptidoglycan structure and membrane lipids contents—which acts as a barrier against penetration of NPs (Hajipour et al. 2012).

Examining the growth curves of *P. syringae* pv. *tomato*, *X. vesicatoria*, *C. michiganensis* subsp. *michiganensis* in the presence of ARGIRIUM‑SUNCs, we noted early and very slight increments of the growth in the presence of the ARGIRIUM‑SUNCs with respect to the control curves. This prompted us to plan experiments for exploring the first 6 h of bacterial growth in the presence of low doses of ARGIRIUM‑SUNCs, corresponding to the EC_10_ and EC_20_ values. The growth curves of *P. syringae* pv. *tomato*, *X. vesicatoria*, *C. michiganensis* subsp. *michiganensis* treated with ARGIRIUM‑SUNCs did not show the lag phase, suggesting that low doses of ARGIRIUM‑SUNCs stimulated early bacterial growth. There is ample evidence that bacteria growth can be stimulated when the sublethal concentration of antimicrobial agents are applied, a phenomenon known as hormesis (Davies et al. 2006; Calabrese 2014), which is also documented in bacteria treated with AgNPs (Fabrega et al. 2009; Sheng and Liu 2011; Xiu et al. 2012; Yang et al. 2013).

We also reported that ARGIRIUM‑SUNCs inhibit biofilm formation for all the phytopathogenic bacteria tested and provoke biofilm eradication for *P. syringae* pv. *tomato*, *X. vesicatoria*, *C. michiganensis* subsp. *michiganensis*. Biofilms play an important role in bacterial plant diseases in both epiphytic and endophytic phases as they protect bacteria from abiotic and biotic stresses such as UV radiation, pH fluctuations, osmotic stress, and dehydration, as well as from antimicrobial agents, antibiotics, defense substances, and toxic compounds released by the host (Bogino et al. 2013; Mina et al. 2019). Consequently, the virulence of many phytopathogenic bacteria is related to the biofilm production, including the bacteria here investigated (Chalupowicz et al. 2012; Killiny et al. 2013; Ichinose et al. 2013; Felipe et al. 2018).

*X. fastidiosa*, which infects many plants, switches its phenotype from a planktonic state to the biofilm aggregation, favoring host colonization and the acquisition by the insect vectors (Landa et al. 2022). The role of biofilm is particularly important in olive quick decline syndrome caused by the xylem-living bacterium *X. fastidiosa* subsp. *pauca*, whose main symptoms seem to be the result of blockage of the plant xylem vessels (D’Attoma et al. 2019). The production of biofilm by *X. fastidiosa* subsp. *pauca*, associated with bacterial multiplication, the production of gums and tyloses by the plant as defense mechanisms, could contribute to the vessel obstructions (D’Attoma et al. 2019).

The ability of ARGIRIUM‑SUNCs in inhibiting both the *X. fastidiosa* subsp. *pauca* growth and the biofilm production would be beneficial in protecting olive plants from (i) the extensive bacterial colonization (planktonic phase); (ii) the biofilming phase, which contributes to the plant symptoms development and to the bacterial spread through vector acquisition. Previously, the antibacterial efficacy of the pesticide Fosetyl-Al (Fos) with respect nanoparticles of Fosetyl-Al (nanoFos) and of chitosan-based Fosetyl-Al (CH-nanoFos) (where chitosan is a coating agent) was in vitro tested against *X. fastidiosa* subsp. *pauca* and *Xylella fastidiosa* subsp. *fastidiosa* (Baldassarre et al. 2020). The results demonstrate that the CH-nanoFos inhibits more promptly and significantly the planktonic growth with respect to Fos and nanoFos on both *X. fastidiosa* subsp. *pauca* and *X. fastidiosa* subsp. *fastidiosa*. Moreover, Fosetyl-Al nanoparticles resulted in very stable over time and less toxic with respect to the conventional formulation on human keratinocytes cells, HaCat cell line, used as in vitro model to test the toxicity of pesticides. This last aspect highlights that the nanoformulation, even without the coating, reduced toxic effects on non-target cells (Baldassarre et al. 2020).

The marked anti-biofilm ARGIRIUM‑SUNCs activities we observed are similar to those reported against *Enterococcus faecium*, *Staphylococcus aureus*, *Klebsiella pneumoniae*, *Acinetobacter bamannii*, *Pseudomonas aeruginosa*, and *Enterobacter* species (ESKAPE), bacteria present in both clinical and food industrial environments (Molina‑Hernandez et al. 2021).

The greater efficacy of ARGIRIUM‑SUNCs in disrupting biofilm with respect to other commercial nanoparticles suggested that supplementary mechanisms with respect to those well-known described are involved. The characteristics of ARGIRIUM‑SUNCs such as the presence of a stable form of rare oxidation silver states (Ag^2+^ and Ag^3+^), an high surface charge (zeta potential >  − 50 mV), a very small average size and polygonal shape, could explain the effective of this nanomaterial to destructure the biofilm by interfering with the cross-links necessary for its three-dimensional structure (Gasbarri et al. 2021; Molina‑Hernandez et al. 2021).

As concerns the effect of ARGIRIUM‑SUNCs on plant-bacterium interactions, we chose tomato as the host plant and *P. syringae* pv. *tomato* as the pathogen, the agent of the bacterial speck disease. Tomato, because it is infected by 3 of the bacteria (*P. syringae* pv. *tomato*, *X. vesicatoria*, *C. michiganensis* subsp. *michiganensis*) tested in the present study, which provoke on this host plant severe economic losses (Thind 2019). We chose the strain DAPP-PG 215 of *P. syringae* pv. *tomato*, whose genome we have recently sequenced, and on the basis of which we have carried out a transcriptomic study to better understand the antibacterial mechanism of ARGIRIUM‑SUNCs (Orfei et al. unpublished results).

Further studies are in progress to evaluate the in vivo efficacy of ARGIRIUM‑SUNCs against *X. vesicatoria* and *C. michiganensis* subsp. *michiganensis*, while our lab is not authorized to test in planta the quarantine bacterium *X. fastidiosa*.

Foliar applications of ARGIRIUM‑SUNCs at 10 ppm were not phytotoxic and did not protect tomato plants against *P. syringae* pv. *tomato* infections. The failure in protecting the plants could be due to the inability or poor ability of ARGIRIUM‑SUNCs to enter into tomato leaves for killing bacteria and/or the relatively low concentrations used with respect to the very high concentration reported in the literature for foliar applications (Tariq et al. 2022). It has been demonstrated that AgNPs are internalized in *Arabidopsis thaliana* through the stomata and that the absorbed amount was related to the entity of stomatal aperture (He et al. 2022). Similar to the results obtained with ZnNPs in rice plants (Khan et al. 2021), we can hypothesize that ARGIRIUM‑SUNCs are not able to enter through the stomata because it provokes their closure. We, therefore, planned experiments to evaluate the protective effect against tomato bacterial speck disease through root ARGIRIUM‑SUNCs applications. We found that tomato roots tolerated high doses of ARGIRIUM‑SUNCs up to 10 ppm. During the experiments for optimizing root applications, we also found that ARGIRIUM‑SUNCs at low non-toxic concentrations marked tomato root growth in a hormetic manner. These results are similar to those reported in tomato plants by Guzman-Baez et al. (2021).

We also demonstrated that tomato plants treated with ARGIRIUM‑SUNCs absorbed by roots for 4 or 8 days protect the plants against *P. syringae* pv. *tomato* attacks. We can therefore suppose that ARGIRIUM-SUNCs are taken up by tomato roots via apoplastic and symplastic pathways and transported to the leaves through the vascular system (Ma et al. 2010), where they exerted their antimicrobial activity. Besides antimicrobial activity, we cannot exclude that ARGIRIUM-SUNCs are also able to induce resistance in tomato plants. For instance, Jiang et al. (2022) reported that AgNPs are able to directly destroy *P. syringae* pv. *tabaci*, the agent of *Nicotiana* spp. angular leaf spot, as well as induce plant resistance in *Nicotiana benthamiana*.

In conclusion, we here demonstrated in vitro that ARGIRIUM‑SUNCs have a strong (< 1 ppm) antimicrobial activity on planktonic growth and biofilm formation against *P. syringae* pv. *tomato* and the quarantine pathogens *X. vesicatoria*, *X. fastidiosa* subsp. *pauca* and *C. michiganensis* subsp. *michiganensis*. We also demonstrated in vivo that ARGIRIUM‑SUNCs are able to protect tomato plants against *P. syringae* pv. *tomato* attacks and that tomato tolerates ARGIRIUM‑SUNCs doses about tenfold higher respect to those showing antibacterial activity. Therefore the application of ARGIRIUM‑SUNCs to plants represents a promising tool for controlling plant bacterial diseases. Further investigations are necessary to know the action and resistance mechanism in bacteria of ARGIRIUM‑SUNCs through transcriptomic, proteomic, and volatilomic analyses (Ronci et al. 2018; Molina-Hernandez et al. 2021).

## Data Availability

Not applicable.
